# Physicochemical Parameters of Raw Pet Food and Dehydrated Pet Treats Developed from Beef Processing Co-Products

**DOI:** 10.3390/ani12030278

**Published:** 2022-01-23

**Authors:** Marc R. Presume, Rigo F. Soler, Moses E. Chilenje, Jorge L. Sandoval, Luis P. Avila, Laura J. Garner, Robert P. Mason, Eric K. Altom, Charles W. Starkey

**Affiliations:** 1 Department of Poultry Science, Auburn University, Auburn, AL 36849, USA; mrp0067@auburn.edu (M.R.P.); solerrigo2015@gmail.com (R.F.S.); oamoseo@gmail.com (M.E.C.); jls0152@auburn.edu (J.L.S.); luis.avila@uga.edu (L.P.A.); bauerlj@auburn.edu (L.J.G.); 2 Animal Nutrition and Health Division, Balchem Corporation, New Hampton, NY 10958, USA; rmason@balchem.com (R.P.M.); ealtom@balchem.com (E.K.A.)

**Keywords:** pet treats, raw pet food, beef processing co-products, upcycling, sodium alginate, encapsulated calcium lactate, pH, water activity, expressible moisture

## Abstract

**Simple Summary:**

Pet food and treat industries are rapidly growing and are important consumers of protein co-products from the human meat processing industry. Unfortunately, there are some co-products such as liver that can be difficult to handle due to the fact it liquifies when ground. This research attempts to address this issue through the use of protein structure forming food technology (hydrocolloids). These technologies may help produce pet foods and treats with higher acceptability, while allow for upcycling of highly nutritious protein co-products. Additions of one such technology (sodium alginate and encapsulated calcium lactate) demonstrated that inclusions of liver could be increased when used in raw pet foods and dehydrated pet treats without negatively impacting chemical attributes that may adversely impact consumer acceptance.

**Abstract:**

Pet humanization and premiumization of pet foods have led to significant changes in the co-product market, as pet food companies are looking for more profitable protein sources for their products. Co-products such as beef liver (BL) and beef heart (BH) can be combined to generate restructured pet foods rich in vitamins and nutrients. Sodium alginate and encapsulated calcium lactate (ALGIN) can improve the acceptability of meat pieces by transforming them into a singular shape. The objective of this experiment was to assess the physiochemical parameters of co-products for utilization in raw pet foods and restructured pet treats generated from BL and BH by using ALGIN as a structure-forming agent. Results demonstrated increased cooking loss as ALGIN inclusion decreased, but cooking loss decreased as BL proportions increased (*p* = 0.0056). Expressible moisture of raw pet food decreased as ALGIN inclusion increased, but more moisture was released from treats when BL proportions increased (*p* < 0.0001). Increasing ALGIN and BH led to increased water activity of cooked treats (*p* < 0.0001). Thus, we suggest that BL and BH combinations with ALGIN inclusion produces a viable platform for higher inclusions of co-products in pet treats. Additionally, these ingredients improved the finished product quality characteristics of raw pet foods.

## 1. Introduction

Beef processing generates a significant amount of protein co-products [1]. Defined as parts of animals that are not included in dressed carcasses [2], animal co-products can represent up to 44% of cattle live weight [1]. Depending on the location and the culture, meat protein processing co-products may be considered a delicacy, or an undesired material destined for purposes other than human consumption. While co-products are often not included in regular meals, they can be found in traditional dishes in various countries, including the USA [1,3]. Despite being undervalued in many situations, some beef processing co-products are rich in vital nutrients such as protein, fats, vitamins, and minerals [3]. Beef liver (BL) is the most consumed co-product and contains approximately 30% protein, close to that of lean meat [4], and is higher in vitamin A, vitamin B12, and iron concentration [5]. Beef heart (BH) is also a highly nutritious organ that has approximately 17% protein [5] and can provide palatable flavors to pet food products [5,6]. Due to their high nutritional content, some beef processing protein co-products may be developed into highly palatable digestible pet food products that are rich in nutrients and flavors [2,7,8].

Hydrocolloids are polysaccharides and sometimes proteins commonly used in the human food industry to improve appearance, texture, and sensory properties of food products [9]. Proper selection of hydrocolloids is crucial to obtaining the ideal physiochemical properties of restructured food products [10]. Some hydrocolloids form thermo-reversible gels unstable under specific temperature ranges; however, other hydrocolloids, such as alginates, can form a stable, thermo-irreversible gel in presence of calcium ions at an ambient temperature [9,10]. Hydrocolloids have also been utilized in pet nutrition products and may provide additional benefits when incorporating ingredients such as liver, which tends to liquefy when ground [11,12,13]. Dainton and colleagues recently demonstrated that hydrocolloid addition could alter batter consistency and, therefore, impact heat penetration in canned pet food [11].

Pet food and pet treat quality and success in the marketplace are determined by several factors such as palatability, texture, color, and overall appearance [14]. Pet owners may also be looking for organic, all natural, and sustainable products for their pets and these factors may influence buying decisions as well [15,16]. Upcycling of low-value co-products from protein conversion industries into higher-value pet nutrition products promotes sustainability, and this feature may be attractive to some pet owners [16,17]. Assessing finished product attributes is essential as they impact customer quality expectations and purchasing decisions. However, information regarding the use of fresh organ meat co-products and gelling agents, specifically alginate, in pet foods and pet treats is limited. Therefore, this study was conducted to evaluate the physicochemical characteristics of raw pet food and dehydrated pet treats developed from three ground, raw BL, and BH mixtures containing one of two concentrations of ALGIN, a sodium alginate (Ingredient Solutions Inc., Waldo, ME, USA) and encapsulated calcium lactate (Balchem Corp. Inc., New Hampton, NY, USA) protein structure-forming component.

## 2. Materials and Methods

### 2.1. Raw Materials Preparation and Ingredients

Frozen, commercially boxed, food-grade BL and BH were stored at 5 °C overnight and then ground individually with an electrical meat grinder (372 J per s) using a 4.5-mm grinder plate. Ground BL and BH were combined in the following 3 ratios until forming a consistent blend: 25%BL:75%BH, 50%BL:50%BH, and 75%BL:25%BH. Ingredient formulations are presented in Table 1.

### 2.2. Structure-Forming Technology Inclusion

Combinations of sodium alginate (Ingredient Solutions Inc.) and encapsulated calcium lactate (Balchem Corp. Inc.; ALGIN) were included in each of 3 BL:BH combinations at 2 concentrations (1× and 0.5×) to generate final batches for each of 6 treatments. The ALGIN inclusion (1×) was determined based on Food and Drug Administration (FDA) recommendations for human food (1% sodium alginate + 0.85% encapsulated calcium lactate). Formulation of ALGIN inclusions was based on the total BL–BH mixture weight (weight per weight; Table 1). Sodium alginate was added to individual BL–BH mixtures and was mixed until thoroughly blended; after this was completed, encapsulated calcium lactate was slowly incorporated in the same manner.

### 2.3. Stuffing, Storage, and Dehydration of Samples

Each ground mixture was extruded using a commercial stuffer with a jerky attachment (20-mm thick) onto parchment paper, refrigerated at 3 °C for 48 h, and then sliced for further analyses. Forty slices (25.4 mm × 63.5 mm) from each treatment were dehydrated in a KOCH smokehouse (model 350003, Ultrasource USA, Kansas City, MO, USA) at 93 °C for 2.5 h. Each sample was weighed before and after dehydration to determine cooking loss using the formula:

$$\left(\frac{\left(sample\;weight\;before\;dehydration-sample\;weight\;after\;dehydration\right)}{sample\;weight\;before\;dehydration}\right)\times 100.$$

### 2.4. Expressible Moisture

Expressible moisture (EM) measurement was conducted on ten raw samples (25.4 mm × 25.4 mm) from each treatment using the filter-press method [18], with modifications. Raw pet food samples were placed on pre-weighed filter papers (35.0 µm pore size and 75-mm diameter, Ahlstrom Munksjo; Mosinee, WI, USA) and compressed with 5 kg for 5 min (Troemner 5-kg class 1 cylindrical calibration weight). After compression, the raw samples were discarded, and the filter papers were re-weighed. Expressible moisture was calculated as percentage of moisture loss during sample compression by using the formula:

$$\frac{\left(weight\;of\;postpress\;filter\;paper-weight\;of\;prepress\;filter\;paper\right)}{sample\;weight}\times 100$$

### 2.5. Water Activity and pH Measurements

The water activity of raw pet food and dehydrated pet treats was measured on ten replicate samples per treatment using the Aqualab water activity meter (model series 3, METER Grp., Pullman, WA, USA). A direct probe was used to measure pH on ten samples from each treatment using a Hach pH meter (model H170G, HACH Co., Loveland, CO, USA). The instrument was calibrated and standardized using pH 4.00 and pH 7.00 buffer solutions for every batch, and the probe tip was rinsed with deionized water and cleaned between each measurement.

### 2.6. Statistical Analysis

A 2-way analysis of variance was performed using a generalized linear mixed model (GLIMMIX) procedure with the Statistical Analysis Software (SAS) version 9.4 (SAS Institute Inc., Cary, NC, USA), with BL:BH combination, ALGIN inclusion, and the interaction of BL:BH combination and ALGIN inclusion as the main effects. In addition to the ANOVA, a complete pairwise mean comparison analysis was performed using the PDIFF option of SAS, and any means within the tables with different superscripts are different at *p* ≤ 0.05. The BL:BH combination × ALGIN inclusion interaction was significant; therefore, all data are presented in this fashion.

## 3. Results

**Figure 1:**
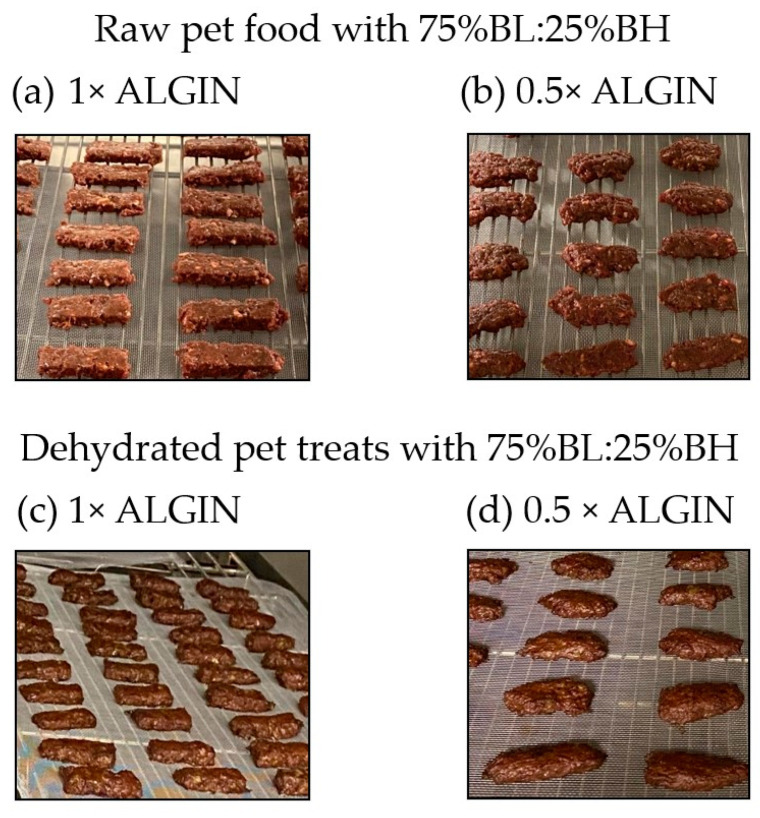
Raw pet food (**a**,**b**) and dehydrated pet treats (**c**,**d**) containing 75% beef liver (BL) and 25% beef heart (BH) and either 1× (1% sodium alginate + 0.85% encapsulated calcium lactate) or 0.5× (0.5% sodium alginate + 0.425% encapsulated calcium lactate) ALGIN exhibit visible differences in three-dimensional structure.

The impact of BL:BH combination and ALGIN inclusion on the physicochemical characteristics of raw pet food and dehydrated pet treats is presented in Table 2. An interaction among BL:BH combination and ALGIN inclusion was observed for all variables, except pH and water activity of the raw pet food. Increasing ALGIN inclusion from 0.5× to 1× visibly improved the three-dimensional structure of both the raw pet food and dehydrated pet treats (Figure 1).

### 3.1. Cooking Loss and Expressible Moisture

Cooking loss was greatest in treats with 50%BL:50%BH + 0.5× ALGIN inclusion. Regardless of BL:BH ratio, as ALGIN inclusion increased, cooking loss decreased (*p* = 0.0056). In treats with 1× ALGIN, increasing BH concentration from 25 to 75%, increased cooking loss. The expressible moisture of the raw pet food decreased as ALGIN inclusion increased, but more moisture was liberated when BL proportions increased (*p* < 0.0001).

### 3.2. Water Activity and pH

The water activity of the raw pet food was greatest in the samples with 25%BL:75%BH + 0.5× ALGIN and lowest in those with 75%BL:25%BH + 1× ALGIN. The water activity of the dehydrated pet treats increased as ALGIN inclusion increased for the 50%BL:50%BH combination and decreased in the 25%BL:75%BH but was not altered in the 75%BL:25%BH combination (*p* < 0.0001). The pH of the raw pet food was unaffected by ALGIN inclusion (*p* = 0.7321) and BL–BH combinations (*p* = 0.8905).

## 4. Discussion

### 4.1. Cooking Loss and Expressible Moisture

Cooking alters meat structural components and water composition. As temperature increases, the meat protein network denatures, water-holding capacity decreases, and proteins contract to expel water [19]. However, addition of hydrocolloids such as alginate can reduce cooking loss [20]. This is likely due to the calcium ions binding with the alginate chains to form junction zones creating a gel [21,22]. In this study, increasing ALGIN hydrocolloid inclusion in BL:BH organ meat mixtures resulted in reduced cooking losses and expressible moisture of dehydrated treats and raw pet food, respectively. Although encapsulated calcium lactate was incorporated in our formulation, these findings are in consistent with other work investigating the use of hydrocolloids, specifically alginate, in restructured meat products [23,24,25,26]. As ALGIN addition increased from 0.5× to 1×, expressible moisture of raw pet food improved regardless of the BL–BH combination, indicating that the water binding capacity of beef organ meat co-product mixtures can be improved by hydrocolloid addition.

Though BH moisture content is typically 7 to 9% greater than BL, organ meat mixtures containing 25% BH and 0.5× ALGIN expelled 7% more moisture compared with those comprising 75% BH. This finding indicates additional water binding capacity of the ground cardiac muscle tissue. It has been suggested in the literature that the lower electroconductivity found in BH may explain this discrepancy in the ability of this particular product to hold moisture under force [7,8]. In addition, it is known that BH is less tender than the BL and may be less likely to express water under external forces due to structural differences in the ground tissues themselves [7,27].

### 4.2. Water Activity and pH

Water activity was highest in raw pet food samples containing 25%BL:75%BH + 0.5× ALGIN and lowest in those with 75%BL:25%BH + 1× ALGIN. The dehydrated treats with 0.5× ALGIN and 75% BH had the highest water activity while those with 50%BL:50%BH + 0.5× ALGIN had the lowest. The interaction among the combination of ground BL–BH and ALGIN inclusion led to inconsistency in the water activity of dehydrated pet treats in this study. Thus, consistent decreases in water activity as ALGIN inclusion increased were not observed in this study as expected. Therefore, the current findings do not align with those that have reported increased water activity in semi-dried chicken jerky products as konjac hydrocolloid inclusion increased [28]. However, the differences in hydrocolloid sources may account for this difference.

Though the water activity values of the dehydrated treats are in a range (>0.9 a_w_) that may be favorable for microbial growth and reduce shelf life, it is also possible that increasing the length of the dehydration period could likely alleviate such issues [29]. Further studies would be necessary to determine if this is the case and how concentrations of BL, BH, and ALGIN may impact the final treat water activity.

The pH of the raw pet food product ranged from 6.29 to 6.33 and was unaffected by both BL–BH combination and ALGIN inclusion. This finding agrees with that of others who demonstrated that inclusion of alginate in restructured meat products did not alter pH [20,24].

## 5. Conclusions

Addition of a structure forming agent (ALGIN, a combination of sodium alginate and encapsulated calcium lactate) to ground BL:BH mixtures at 1× compared with 0.5× visibly improved the three-dimensional product structure of both raw food and dehydrated treats, increased water retention of raw pet food, and increased final water activity of cooked pet treats. Importantly, addition of this particular restructuring agent permitted the inclusion of 75% ground BL, which essentially liquifies upon grinding, thus demonstrating that lower value beef processing co-products can be utilized to develop pet food and pet treat products. Moving forward, it will be important to evaluate the organoleptic attributes and acceptance by both pets and pet owners in future studies.

## Figures and Tables

**Table 1:** Formulations of beef liver (BL) and beef heart (BH) combinations and sodium alginate + encapsulated calcium lactate (ALGIN) inclusions utilized in the manufacture of pet nutrition products.

	Treatments					
Ingredients	Trt 1	Trt 2	Trt 3	Trt 4	Trt 5	Trt 6
Beef liver, %	25	25	50	50	75	75
Beef heart, %	75	75	50	50	25	25
Total	100	100	100	100	100	100
Sodium alginate, %	1.0	0.5	1.0	0.5	1.0	0.5
Encapsulated calcium lactate, %	0.85	0.425	0.85	0.425	0.85	0.425

**Table 2:** Effect of beef liver (BL) and beef heart (BH) combination and sodium alginate + encapsulated calcium lactate (ALGIN) inclusion on cooking loss, expressible moisture, water activity, and pH of raw pet food and dehydrated pet treats.

Variable	Combination of Beef Liver (BL) and Beef Heart (BH) ^1^						SEM ^3^	*p-*Value
	25%BL:75%BH		50%BL:50%BH		75%BL:25%BH			
	ALGIN Inclusion, % ^2^							
	0.5×	1×	0.5×	1×	0.5×	1×		
Cooking loss, %	45.96 ^b^	43.66 ^c^	47.55 ^a^	42.54 ^dc^	45.23 ^b^	41.61 ^d^	0.4361	0.0056
Expressible moisture, %	10.77 ^b^	7.33 ^d^	11.35 ^b^	8.78 ^c^	17.91 ^a^	9.22 ^c^	0.7148	<0.0001
Raw pet food water activity, a_w_	1.011 ^a^	1.005 ^ab^	1.007 ^ab^	1.007 ^ab^	1.007 ^ab^	1.002 ^b^	0.0024	0.4347
Dehydrated treat water activity, a_w_	0.980 ^a^	0.963 ^bc^	0.939 ^d^	0.970 ^b^	0.956 ^c^	0.964 ^bc^	0.0036	<0.0001
Raw pet food pH	6.33	6.29	6.32	6.32	6.33	6.33	0.0393	0.8580

^1^ Beef liver (BL) and beef heart (BH) were ground and mixed to achieve the following 3 BL:BH combinations: 25%BL:75%BH, 50%BL:50%BH, and 75%BL:25%BH. ^2^ ALGIN is a structure-forming agent composed of 2 functional ingredients: sodium alginate and encapsulated calcium lactate incorporated during manufacturing at either 1× and 0.5× (1× ALGIN = 1% of sodium alginate and 0.85% of encapsulated calcium lactate). ^3^ SEM = highest standard error of the LS means. ^a–d^ Means with different superscripts differ *p* ≤ 0.05 and were generated from the pairwise mean comparison analysis.

## Data Availability

The data presented in this study are available on request from the corresponding author.
